# A transcriptomic resource for glial GABA-associated ASH neuronal aging and candidate pathways

**DOI:** 10.3389/fnagi.2026.1677754

**Published:** 2026-02-23

**Authors:** Umar Al-Sheikh, Hankui Cheng, Ahmed Abdulsalam Ali Bakrbaldawi, Longyuan He, Du Chen, Renya Zhan, Lijun Kang, Yongming Zhang

**Affiliations:** 1 Department of Ophthalmology of the Fourth Affiliated Hospital and School of Brain Science and Brain Medicine, Zhejiang University School of Medicine, Yiwu, China; 2 Department of Neurosurgery of the First Affiliated Hospital and School of Brain Science and Brain Medicine, Zhejiang University School of Medicine, Zhejiang, China; 3 Liangzhu Laboratory, MOE Frontier Science Center for Brain Science and Brain-Machine Integration, Zhejiang University, Hangzhou, China; 4 State Key Laboratory of Brain-Machine Intelligence, Zhejiang University, Hangzhou, China; 5 NHC and CAMS Key Laboratory of Medical Neurobiology, School of Medicine, Zhejiang University, Hangzhou, Zhejiang, China; 6 Department of Neurosurgery, School of Medicine, The Second Affiliated Hospital of Zhejiang University, Hangzhou, China

**Keywords:** *C. elegans*, GABA, glia-neuronal interaction, neuronal aging, transcriptomic analyses

## Abstract

**Introduction:**

Neuronal aging is tightly linked to neurodegeneration with dysregulation of GABA (gamma-aminobutyric acid), the primary inhibitory neurotransmitter, contributing to age-associated neuronal impairment. Our prior work demonstrated that restoring the key GABA-synthesizing enzyme UNC-25 (glutamic acid decarboxylase, GAD) in *Caenorhabditis elegans* AMsh glia mitigates age-related neurodegeneration. This study aims to provide a transcriptomic resource and identify potential pathways associated with glial GABA modulation during neuronal aging.

**Methods:**

ASH neurons from day 1 and day 7 nematodes were isolated and FACS-purified (Psra-6::RFP+/Pgpa-4::GFP-) from three distinct groups: Wild-type, *unc*-25 mutants, *unc*-25 mutants with AMsh glia-specific UNC-25 rescue. RNA-seq used Illumina NovaSeq (150 bp PE reads, aligned to WormBase WS293). DESeq2 identified DEGs (FDR < 0.05, fold-change ≥ 1); clusterProfiler performed GSEA and pathway enrichment. Comparisons also included AMsh glia vs. ASH neurons in wild young adults.

**Results:**

Here, we present transcriptomic data of glutamatergic ASH sensory neurons (a critical target of aging-related neurodegeneration) from three aging groups: wild-type worms, *unc*-25 (GABA-deficient) mutants, and *unc*-25 mutants with AMsh glia-specific UNC-25 rescue. Transcriptomic analyses revealed distinct transcriptional profiles across groups. Notably, the Hedgehog signaling pathway and its transcriptional effector TRA-1/GLI, the *C. elegans* GLI ortholog, were specifically upregulated in the glial rescue group, while the neuroprotective transcription factor HSF-1 was downregulated, suggesting these pathways as potential mediators of glial GABA-associated neuroprotection. We also provide transcriptomic comparisons between AMsh glia and ASH neurons in young worms, laying a foundation for understanding glia-neuron crosstalk.

**Conclusions:**

This work establishes a valuable transcriptomic resource for glial GABA-associated ASH neuronal aging and identifies candidate pathways, offering critical molecular insights to dissect age-related neurodegeneration mechanisms and inform potential therapeutic targets.

## 1 Introduction

Neuronal aging is a multifaceted intrinsic biological process that significantly impacts lifespan and brain health. Notably, neuronal aging is closely linked to the development of neurodegenerative diseases such as Alzheimer’s, Parkinson’s, and Huntington’s diseases (Rhine et al., 2025). During the aging process, numerous structural and functional changes occur including the loss of neurons, reduced synaptic plasticity, and increased oxidative stress — all of which further contribute to cognitive decline and the progression of neurodegenerative diseases (Hou et al., 2019). These changes result from the complex interplay between aging neurons and glial cells, which play a crucial role in maintaining neuronal health (Rawji et al., 2023). Notably, neural activity itself influences lifespan — excessive neural excitation correlates with shorter lifespans, while suppression of overactivity extends longevity (Wirak et al., 2022).

Gamma-aminobutyric (GAMA) acid is the primary inhibitory neurotransmitter, essential for maintaining neuronal homeostasis and preventing excessive neuronal excitation (Cheng et al., 2024; Wu et al., 2022). However, with aging, there is a notable decline in GABA levels, which leads to excitotoxicity and cell death, contributing to the progression of neurodegenerative disorders (Cheng et al., 2024; Zuppichini et al., 2024). This dysregulation of GABAergic signaling highlights its critical role in preserving neuronal resilience and function. In addition, glial cells, such as astrocytes and oligodendrocytes, are vital for supporting neuronal health by regulating neurotransmitter uptake, synaptic plasticity, and myelination (Nave, 2010). In the context of aging, glial cells also regulate GABAergic signaling, making them integral to maintaining inhibitory neurotransmission (Kenyon, 2010; Singhvi and Shaham, 2019). However, a comprehensive understanding of how glial-derived GABA specifically modulates neuronal aging processes remains incomplete. Gaps persist in our understanding of the molecular pathways through which glial cells orchestrate GABA dynamics to counteract age-related neuronal dysfunction, and how this glia-neuron crosstalk influences neuronal survival and cognitive function.

The nematode *Caenorhabditis elegans* is a powerful model for studying aging and neurodegeneration. Its well-characterized genome, simple nervous system (302 neurons and 56 glial cells), short lifespan, and ease of genetic manipulation make it an excellent model for investigating the molecular mechanisms underlying neuronal aging (Duan et al., 2020). *C. elegans* has been extensively used to study aging-related processes, including stress responses, mitochondrial dynamics, and insulin/IGF-1 signaling pathways — all of which are relevant to neurodegenerative disease mechanisms in humans (Lee and Lee, 2022; Li et al., 2024). The Amphid sheath (AMsh) glia is a crucial structure that envelops sensory receptive endings of most amphid neurons in the nematode head (Singhvi et al., 2024). The AMsh glia either forms the amphid channel through which neuronal cilia extend or directly embeds dendritic endings within the glial sheath. Among the amphid neurons, the polymodal ASH sensory neurons are critical modulators of lifespan through neural activity, neuropeptide secretion, and sensory signaling (Cheng et al., 2024; Duan et al., 2020).

Given that *C. elegans* has a simple and conserved neural structure, we focused on AMsh glia and ASH sensory neurons in the amphid sensory organ of its head — their intercellular communication is crucial for maintaining the function of sensory neurons. Besides, AMsh glia and ASH neurons form a critical interface for glia-neuronal crosstalk in the amphid sensory organ, particularly influencing the function and longevity of ASH sensory neurons. Notably, aging ASH neurons communicate with AMsh glia by transmitting heat shock protein HSP-4 via extracellular vesicles, thereby activating the glial unfolded protein response pathway (IRE1-XBP1) — a key protective stress response that enhances glial support for neurons (Wu et al., 2025). This glial response enhances protective mechanisms by upregulating chondroitin synthases, contributing to extracellular matrix remodeling that maintains neuronal function and delays sensory decline. Thus, AMsh glia serve as an essential mediator of glia-neuron signaling that influences neuronal aging and sensory integrity in *C. elegans*.

Building on our previous work demonstrating that AMsh glia modulate ASH neuronal aging via bestrophin-dependent GABA release acting on the GABA_*B*_ receptor GBB-1, the molecular underpinnings mediating the impact of AMsh glial GABA on ASH neuronal aging remain poorly defined. In this study, we employed single-cell transcriptomics to systematically profile transcriptional changes in ASH neurons during aging and characterize the transcriptomic signatures associated with glial GABA modulation. Specifically, our research focused on generating a transcriptomic resource for glial GABA-associated ASH neuronal aging and identifying potential molecular pathways linked to this interaction. By dissecting the transcriptomic alterations in ASH neurons across wild-type, *unc-25* mutant, and glial *unc-25*-rescued animals, our goal is to map the transcriptional dynamics in aging neurons correlated with glial GABA and uncover candidate signaling cascades that may contribute to neuroprotection or neurodegeneration.

## 2 Materials and methods

### 2.1 *C. elegans* strains

The strains were maintained under standard conditions at 20°C on nematode growth medium (NGM) plates, which were seeded with the OP50 strain of *Escherichia coli*. L4 larvae hermaphrodites were transferred to fresh NGM plate, and the next day their “age” was counted as day 1 of adulthood. Day 7 worms were considered as aged worms, and day 1 worms were used as control, unless specified otherwise. Worms were well-fed and transferred to another NGM plate every 2 days (Cheng et al., 2024).

The *C. elegans* strains utilized in this study are listed below:

(1) N2; *KanEx1049[Pgpa-4::GFP+Psra-6::RFP]*
(2) *unc-25(e156);KanEx1049[Pgpa-4::GFP+Psra-6::RFP]*
(3) *unc-25(e156);KanEx1050[Pgpa-4::GFP+Psra-6::RFP+Pvap-1::unc-25(cDNA)::sl2::BFP]*

### 2.2 Primary cell isolation

To prepare the isolated ASH neurons, a method previously described was followed (Spencer et al., 2014). Gravid adults were maintained on peptone-enriched plates (8P) seeded with *E. coli* OP50. Eggs were isolated, concentrated, and transferred to NGM plates with OP50 for synchronized growth to either Day 1 or Day 7 adults. Synchronized adults underwent enzymatic digestion to generate dissociated cell preparations (Imanikia et al., 2019). Prior to sorting, cell suspensions were filtered through a 5-μm nylon mesh to remove aggregates.

### 2.3 Fluorescence-activated-cell-sorting (FACS)

Amphid Single Cilium H (ASH) neurons were identified via *Psra-6::RFP* expression. To exclude ASI neurons contaminating the initial sort, Fluorescent cells were re-sorted based on the absence of *Pgpa-4::GFP* signal. Purified ASH neurons were collected directly in TRIzol LS reagent (Thermo Fisher) for RNA stabilization and subsequent extraction. RNA sequencing libraries were prepared and deep-sequenced by a commercial service provider.

### 2.4 RNA sequencing analysis

After library quality assessment, sequencing was performed on the Illumina NovaSeq platform, generating 150 bp paired-end reads. To ensure data quality and reliability, raw reads were filtered, and all downstream analyses utilized high-quality clean reads. A reference genome index was constructed using HISAT2 v2.2.5, and paired-end reads were aligned to the reference genome. The reference genome and gene annotation files were obtained from WormBase (WS293); genes annotated as “Dead” were excluded, retaining only those labeled as “Live.” Gene-level read counts were quantified using FeatureCounts v1.5.0-p3, and gene FPKM values were calculated based on gene length and mapped read counts. Following sequencing, the provider supplied gene and transcript count matrices after processing with Cutadapt, Trimmomatic, FastQC, HISAT2, and StringTie.

Initial assessment revealed a substantial proportion of zero counts across samples. To improve statistical power, an additional biological replicate was generated for the wild-type day 1 group used in ASH vs. AMsh day 1 analysis. To account for zero inflation and technical dropouts inherent in the count data, we applied ZINB-WaVE (version 1.3.0) prior to downstream analysis (Risso et al., 2018). Differential expression analysis was performed using DESeq2 (version 1.4.1) on the ZINB-WaVE output (He et al., 2021).

Benjamini and Hochberg method was used to obtain the false discovery rate (FDR) from *P*-values. As a criterion for significantly differential expression, a corrected value of FDR < 0.05 and Fold change ≥ 1 was chosen. The clusterProfiler R package was used to perform the gene ontology (GO) enrichment analysis of differentially expressed genes (DEGs) with an adjusted value of *P* < 0.05. Fisher’s exact test was also applied to these DEGs lists to identify gene sets that significantly overlapped with the Kyoto encyclopedia of genes and genomes (KEGG) pathway gene sets. Additional enriched pathways of GO, KEGG and Reactome were identified using The Database for Annotation, Visualization, and Integrated Discovery (DAVID).

GSEA was performed by ranking all genes based on their level of difference using R package clusterProfiler (version 4.16.0) and visualized using R package enrichplot (version 1.28.2). Heatmap were visualized using ggplot2 (version 3.5.2) and pheatmap (version 1.0.13).

## 3 Results

### 3.1 Transcriptomic alterations in aging ASH neurons

*C. elegans*, similar to other animals and humans, undergoes functional and structural decline during aging (Zhang et al., 2020). Due to highly conserved genetic pathways and homologies with humans, nematodes serve as an important model to study the mechanisms of neuronal aging (Van Pelt and Truttmann, 2020). To investigate the age-dependent transcriptomic changes in ASH neurons, we focused on the wild-type worms at day 1 (D1) and day 7 (D7). The D1 worms represent the onset of adulthood with peak physiological functions, while D7 adults correspond to a late stage characterized by cellular and functional declines, marking the progression of aging (Pincus et al., 2011). Transcriptomic analyses disclosed that aging in *C. elegans* is associated with widespread changes in gene expression, signaling pathways, and cellular functions (Schiksnis et al., 2024; Wang X. et al., 2022).

**FIGURE 1:**
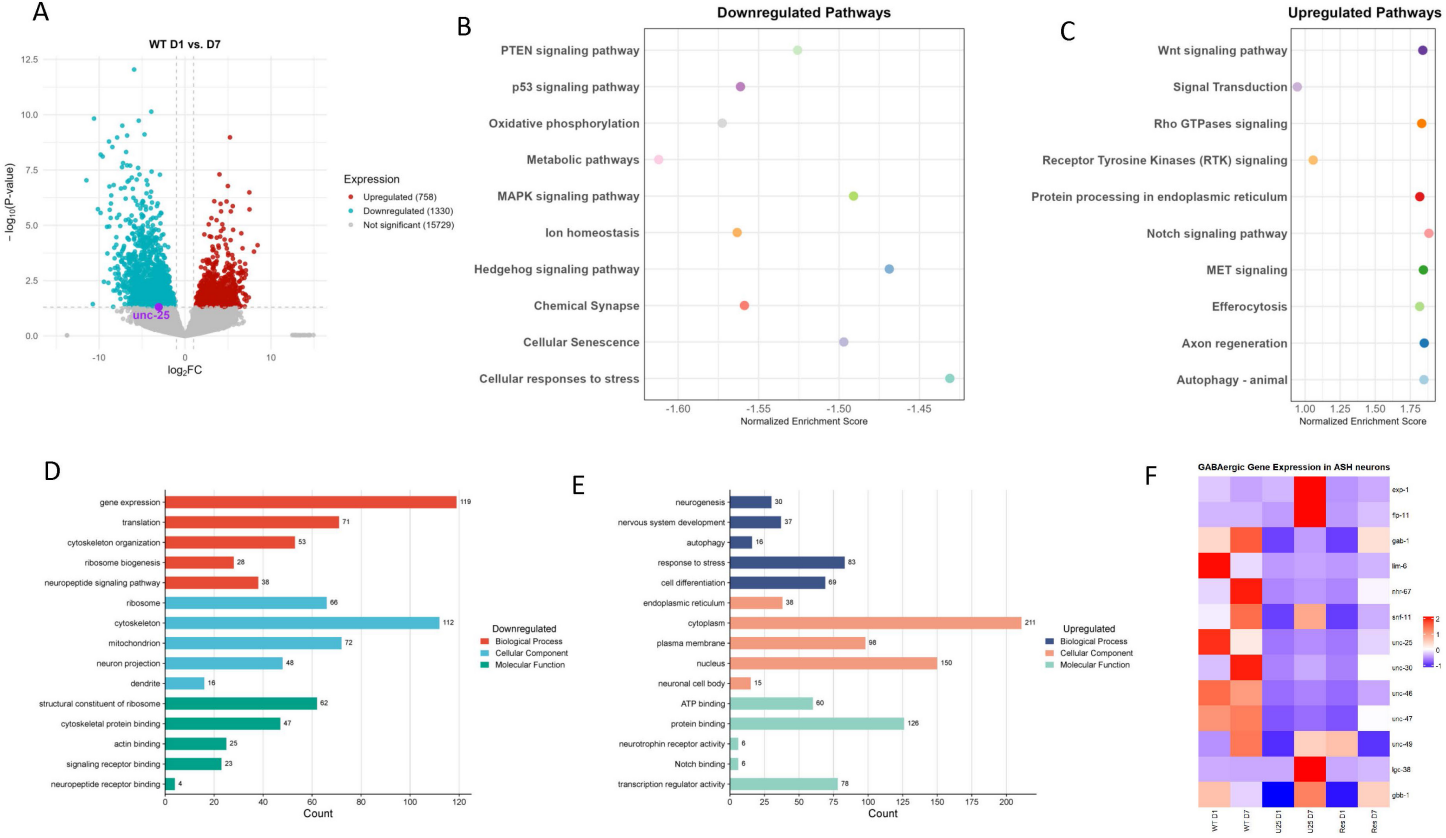
**(A)** Volcano plot of wild-type (WT) day 1 (D1) vs. day 7 (D7). **(B)** Downregulated pathways. **(C)** Upregulated pathways. **(D)** Downregulated GO terms. **(E)** Upregulated GO terms. **(F)** GABAergic gene expressions in ASH neurons.

Notably, we observed a significant downregulation of *unc-25*, a key gene for GABA synthesis, predominantly at D7, as revealed by differential expression analysis (Figure 1A). Age-associated loss of inhibitory GABAergic signaling has been reported to disrupt the crucial excitatory/inhibitory balance and contributes to neuronal functional decline (Wirak et al., 2022). Furthermore, we detected numerous GABAergic gene expression changes in aging ASH neurons, highlighting broad alterations in inhibitory signaling (Figures 1A–F). Downregulation of oxidative phosphorylation, metabolic pathways, and mitochondrial activity at D7 represents a hallmark of neuronal aging and neurodegeneration (Anson and Hansford, 2004). Concurrent downregulation of pathways involved in cellular senescence, ion homeostasis, chemical synapse function, and neuropeptide signaling further indicates reductions in synaptic activity, structural maintenance, and stress responsiveness (Figure 1B).

Conversely, upregulated pathways at D7 included efferocytosis, Wnt signaling, axon regeneration, and autophagy (Figure 1C). Enrichment of GO terms related to neurogenesis, nervous system development, and autophagy highlights the activation of compensatory and regenerative processes, representing adaptive responses to age-related decline (Figures 1D, E). The upregulation of Notch, Receptor Tyrosine Kinases (RTK), and Rho GTPase signaling pathways further implicates engagement of conserved developmental and signaling cascades that modulate neuronal maintenance and plasticity in aging nervous system. However, the Hedgehog signaling pathway was significantly downregulated with age; since this pathway is essential for sustaining adult neurogenesis — including the maintenance and generation of GABAergic neurons — its decline may exacerbate GABAergic dysfunction and contribute to increased neuronal vulnerability during aging (Figure 1B; Parashar et al., 2024; Wang J. et al., 2022; Yang et al., 2021).

Moreover, the increased expression of genes involved in protein processing within the endoplasmic reticulum, along with transcription regulator activity, indicates heightened proteostatic and transcriptional regulation, reflecting cellular adaptations to accumulated stress (Taylor and Hetz, 2020; Zhang et al., 2022). Together, these findings highlight a complex molecular interplay in aging ASH neurons, characterized by diminished metabolic and synaptic functions coupled with enhanced regenerative signaling pathways — such as Notch — that may serve as intrinsic mechanisms to counteract neurodegenerative processes (Naranjo-Galindo et al., 2022).

### 3.2 GABA loss and neuronal aging

**FIGURE 2:**
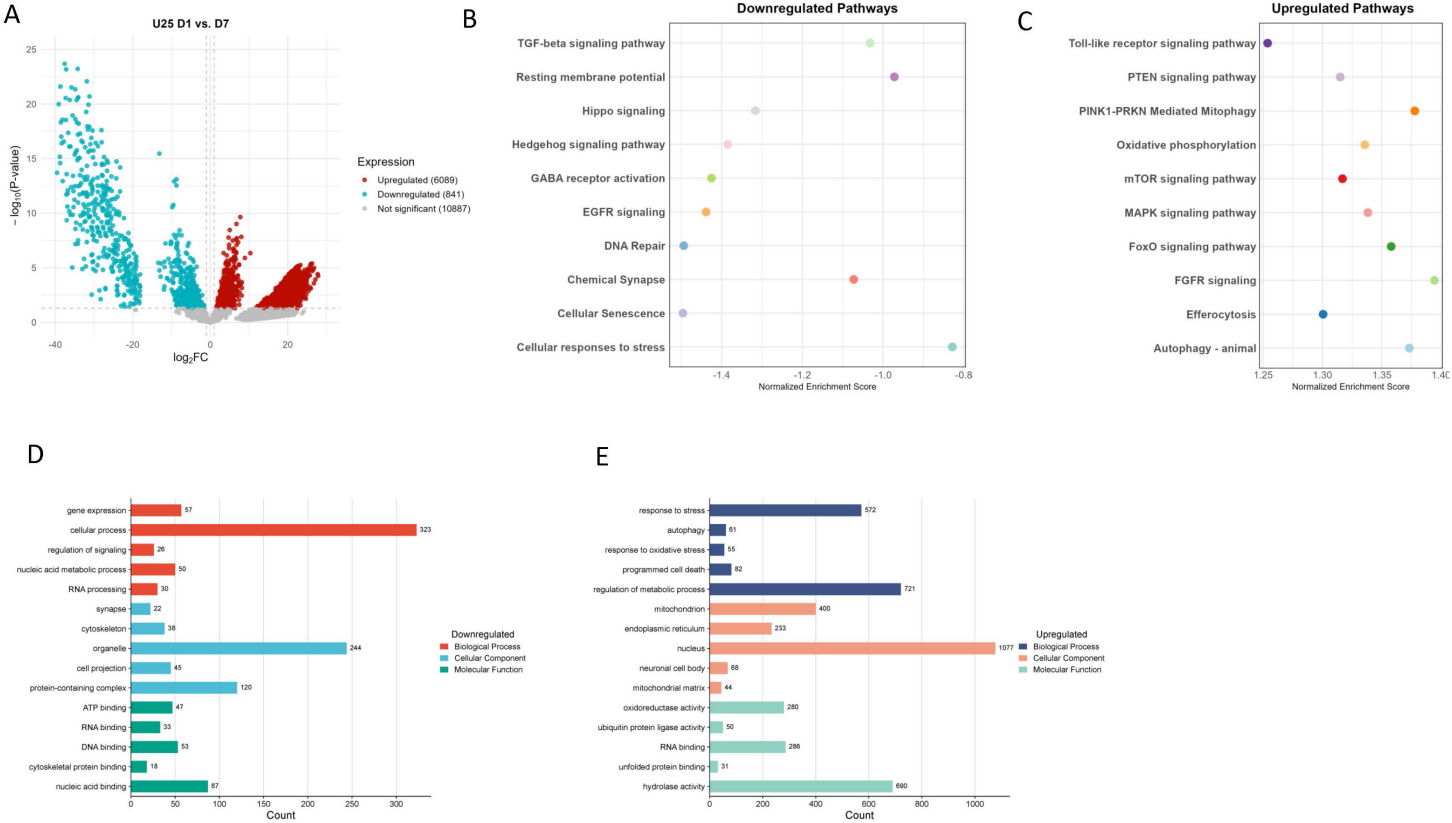
**(A)** Volcano plot of UNC-25 mutant (U25) day 1 (D1) vs. day 7 (D7). **(B)** Downregulated pathways. **(C)** Upregulated pathways. **(D)** Downregulated GO terms. **(E)** Upregulated GO terms.

To investigate the role of GABA in ASH neuronal aging, we examined transcriptomic changes of *unc-25* mutants, which lack functional glutamate decarboxylase (GAD). GAD dysfunction has significant implications for a range of neurological disorders due to its critical role in synthesizing GABA (Lange et al., 2014). We compared *unc-25* mutants to wild-type at both day 1 (D1) and day 7 (D7) to assess immediate and progressive effects of GABA loss on age-associated molecular changes (Figure 2A). We found that loss of GABA in *unc-25* mutants leads to upregulation of pathways associated with mitochondrial function and cellular maintenance mechanisms, including oxidative phosphorylation, FoxO and mTOR signaling, efferocytosis, autophagy, mitophagy, Toll-like receptor signaling, and MAPK pathways (Figures 2B, C). This pattern reflects heightened activation of stress responses and catabolic mechanisms, acting as compensatory adaptations to mitochondrial dysfunction and increased cellular stress in aging neurons deficient in GABA synthesis.

Conversely, *unc-25* mutants exhibit downregulation of critical neuronal and signaling pathways, such as chemical synapse, Hippo and Hedgehog signaling, resting membrane potential regulation, DNA repair, EGFR signaling, and GABA receptor activation (Figures 2B, C). Notably, the suppression of Hedgehog and Hippo pathways — both established regulators of neurogenesis and synaptic plasticity — suggests an accelerated decline in regenerative and neuronal maintenance capacity in the absence of GABA (Knowles et al., 2014). This overall downregulation signals progressive synaptic dysfunction, impaired neuronal communication, and reduced cellular repair during aging. Gene Ontology (GO) analysis further supports with downregulated terms involving gene expression, nucleic acid metabolism, RNA processing, synapse structure, cytoskeletal organization, and nucleic acid/protein binding, together indicating broad impairment in transcriptional activity and neuronal architecture (Figure 2D). In contrast, upregulated GO terms include responses to stress and oxidative stress, autophagy, programmed cell death, mitochondrial and endoplasmic reticulum functions, and proteostasis mechanisms — highlighting intensified cellular stress management and degradation responses, consistent with neurodegenerative stress adaptation (Figure 2E; López-Otín et al., 2013; Ye et al., 2023).

Together, these results underscore that loss of GABA synthesis due to *unc-25* mutation exacerbates age-associated neurodegenerative processes by disrupting synaptic signaling and neuronal communication, while triggering increased stress responses and mitochondrial activation. The downregulation of Hedgehog and GABA receptor pathways in particular suggests impaired regenerative capacity and inhibitory neurotransmission, thereby accelerating neurodegeneration in aging *unc-25* ASH neurons. Previously, we observed that loss of GABA (*unc-25*) significantly accelerates neurodegeneration in aging worms (Cheng et al., 2024).

**FIGURE 3:**
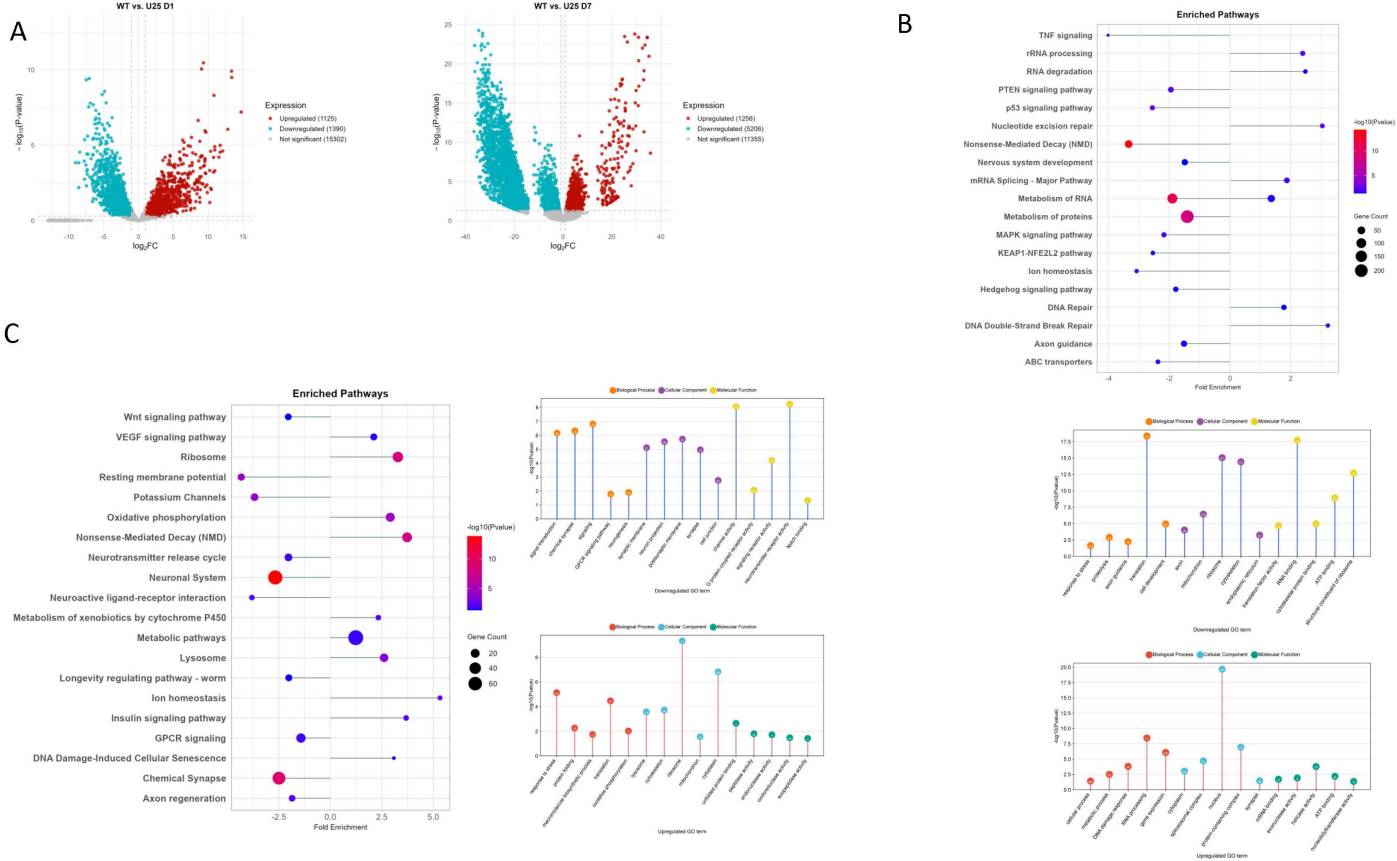
**(A)** Volcano plots of wild-type (WT) vs. UNC-25 (U25) at day 1 (D1) and day 7 (D7). **(B)** Enriched pathways and GO terms for WT vs. U25 D1. **(C)** Enriched pathways and GO terms for WT vs. U25 D7.

We next compared Wild-type and UNC-25 mutation at D1 and found profound transcriptomic changes that shed light on the molecular consequences of GABA loss and its relation to aging processes. In *unc-25* mutants, a substantial number of genes are differentially altered, indicating widespread alterations in cellular function (Figure 3A). Among the downregulated pathways, several key signaling cascades such as p53 signaling, PTEN signaling, MAPK signaling, TNF signaling, and KEAP1-NFE2L2 pathway are notably suppressed (Figure 3B). These pathways are crucial for maintaining cellular stress responses, survival, and neuronal development, suggesting that GABA deficiency disrupts essential mechanisms that normally support neuronal health and longevity. The observed downregulation of protein metabolism, nonsense-mediated decay, translation machinery components (ribosome, translation factors), and cellular structures like mitochondria and cytoskeleton further points to impaired cellular homeostasis and protein synthesis, which are hallmarks of aging and neurodegeneration (Koh et al., 2025; Ma et al., 2024).

Moreover, genes involved in ion homeostasis, axon guidance, and nervous system development are significantly reduced in expression. This pattern implies that GABA loss accelerates early aging-like decline by weakening the ability of neurons to maintain stability, guidance, and functional integrity. On the other hand, pathways related to DNA repair (nucleotide excision repair, DNA double-strand break repair), RNA processing (RNA degradation, rRNA processing, mRNA splicing), and metabolic processes are upregulated, indicating an enhanced cellular response to genomic and transcriptomic stress (Figure 3B). This suggests that *unc-25* mutants trigger compensatory mechanisms aiming to repair DNA damage and maintain RNA stability, consistent with cellular stress and damage responses observed during the early stages of neurodegenerative processes (Madabhushi et al., 2014; Ye et al., 2023). Gene ontology analysis further supports these findings by showing increased activity in processes related to DNA damage response, RNA processing (spliceosomal complex, mRNA binding), and nucleotide-processing enzymatic activities (exonuclease and helicase activity). These changes may represent an attempt by neurons to counteract early damage induced by loss of inhibitory GABA signaling and maintain cellular function.

At day 7, the transcriptomic profile of *unc-25* mutants highlights progressive neuronal aging and dysfunction driven by sustained GABA loss. A large number of genes are downregulated, reflecting a shift in cellular responses over time (Figure 3A). Positively enriched pathways such as oxidative phosphorylation, nonsense-mediated decay (NMD), lysosome function, ion homeostasis, insulin signaling, and DNA damage-induced cellular senescence indicate that ASH neurons in *unc-25* mutants are increasingly activating stress response and cellular proteostasis networks to cope with accumulated damage (Figure 3C). The upregulation of oxidative phosphorylation represents a compensatory effort to maintain energy production despite mitochondrial stress commonly observed in aging neurons (Ye et al., 2023).

Conversely, critical pathways involved in neuronal support and maintenance — axon regeneration, Wnt signaling, longevity regulating pathway, neuroactive ligand-receptor interaction, neuronal system, chemical synapse, and GPCR signaling — are negatively enriched, suggesting a decline in neuroprotective and regenerative capacity (Figure 3C). The suppression of axon regeneration and synaptic pathways is particularly relevant, as these processes are essential for maintaining neuronal connectivity and plasticity (Byrne and Hammarlund, 2017). Downregulated GO terms include neurogenesis, neuron projection, synapse, synaptic membrane, postsynaptic membrane, and cell junction, indicating impaired neuronal structure and signaling — hallmarks of neuronal aging and decline (Castelli et al., 2019).

The prolonged GABA deficiency exacerbates age-associated neuronal dysfunction by impairing regenerative and homeostatic pathways while further activating stress responses such as protein folding, unfolded protein binding, and lysosomal degradation. This shift from early metabolic impairment and DNA repair (observed at D1) toward sustained stress, cellular senescence, and inflammatory-like responses suggests that GABA deficiency accelerates the molecular hallmarks of neuronal aging, including progressive loss of regenerative capacity and increased cellular stress, which are key contributors to late-stage neuronal dysfunction (Drumond-Bock et al., 2025; Loerch et al., 2008; Rozycka and Liguz-Lecznar, 2017). Notably, the downregulation of Notch binding-related pathways at day 7 highlights an additional layer of impaired signaling important for neuroprotection, synaptic plasticity, and neuronal survival. Notch signaling in mature neurons supports adaptive synaptic remodeling and cellular stress resistance, and its suppression may contribute to reduced neuronal resilience and functional decline (Kim et al., 2015; Wisniewski et al., 2011).

### 3.3 GABA rescue in adjacent AMsh glia

**FIGURE 4:**
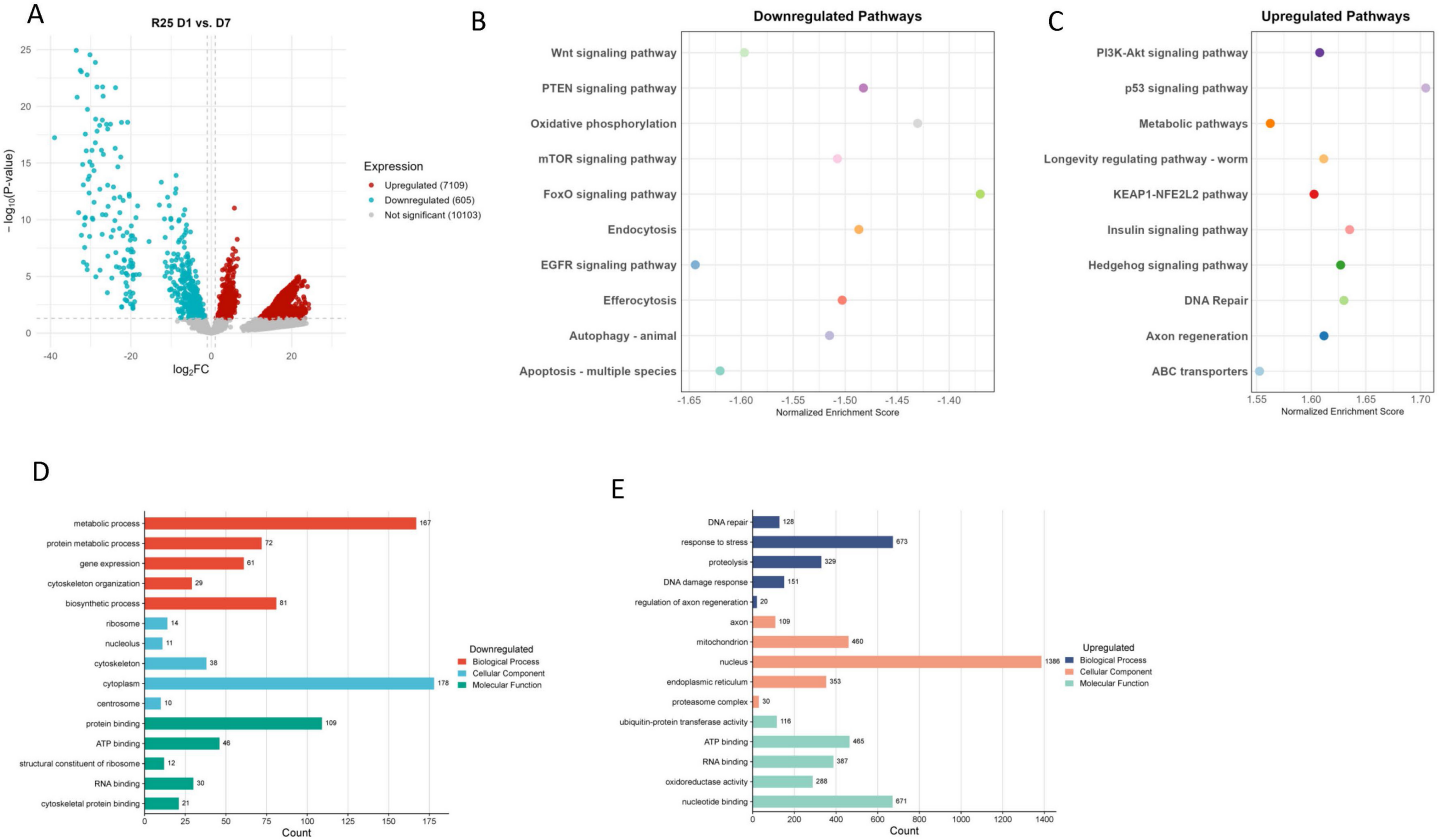
**(A)** Volcano plot of UNC-25 rescue (R25) day 1 (D1) vs. day 7 (D7). **(B)** Downregulated pathways. **(C)** Upregulated pathways. **(D)** Downregulated GO terms. **(E)** Upregulated GO terms.

We next restored *unc-25* expression in AMsh glia, which likely reinstates proper GABAergic signaling, essential not only for synaptic inhibition but also for maintaining neuronal homeostasis during aging (Figure 4A). Following rescue, we observed a prominent downregulation of metabolic and biosynthetic pathways, including oxidative phosphorylation, mTOR signaling, FoxO signaling, autophagy, and apoptosis, indicating a shift toward reduced energy consumption, altered programmed cell death, and cellular clearance mechanisms at day 7 (Figure 4B). This coordinated reduction in energy-demanding processes and apoptotic signaling may help protect neurons from metabolic exhaustion and inappropriate cell loss, reflecting an adaptative strategy to maintain neuronal viability and function during aging. Such response is an established protective hallmark observed in neurodegenerative models, where metabolic attenuation and controlled suppression of apoptosis can limit neuronal vulnerability and promote cellular longevity (Lin and Beal, 2006; Mattson and Arumugam, 2018).

Conversely, key pathways such as the Hedgehog signaling, DNA repair, p53 signaling, PI3K-Akt signaling, insulin signaling, axon regeneration, and KEAP1-NFE2L2 antioxidant response pathway were upregulated, reflecting activation of protective stress responses, DNA damage repair, survival, and regenerative programs (Figure 4C). The induction of DNA repair and antioxidant defenses is essential for counteracting accumulated cellular insults during aging, thereby supporting genomic stability and protein homeostasis. This shift parallels findings in other systems where the upregulation of stress response pathways, coupled with regenerative programs, enhances neuroprotection and promotes recovery from aging-related decline (Dinkova-Kostova et al., 2018; López-Otín et al., 2013). These pathway changes are complemented by upregulated GO terms related to DNA repair, response to stress, proteolysis, mitochondrial function, and protein ubiquitination, supporting enhanced cellular maintenance and damage mitigation processes (Figures 4D, E).

Furthermore, activation of axon regeneration and longevity-regulating pathways suggests that ASH neurons with *unc-25* rescue engage in repair and maintenance programs that may actively counteract age-associated functional decline. These neuroprotective mechanisms not only support recovery from existing damage but may also endow neurons with greater resilience to future stressors, a key feature of healthy aging (Chung et al., 2016; Saijilafu Zhang and Zhou, 2013). The combined effects of metabolic downregulation and upregulation of stress and repair pathways provide compelling evidence for a protective transcriptional reprogramming that fosters neuronal resilience. Therefore, glial expression of *unc-25* not only restores GABA production but also triggers broad neuroprotective and regenerative transcriptional programs, including Hedgehog signaling, eventually promoting neuronal homeostasis, maintenance, and functional recovery during aging (Figures 4D, E).

**FIGURE 5:**
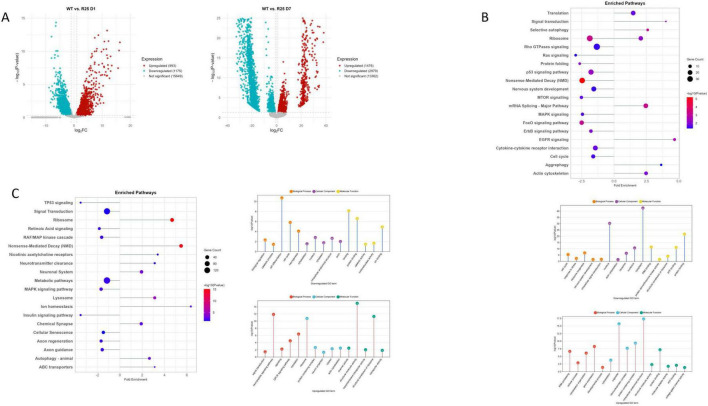
**(A)** Volcano plots of wild-type (WT) vs. UNC-25 rescue (R25) at day 1 (D1) and day 7 (D7). **(B)** Enriched pathways and GO terms for WT vs. R25 D1. **(C)** Enriched pathways and GO terms for WT vs. R25 D7.

To further examine these processes, we compared wild-type and UNC-25 rescue at D1, which revealed robust activation of pathways critical for neuronal remodeling and cellular maintenance (Figure 5A). The rescue enhances signaling cascades such as EGFR signaling and signal transduction, which are well known to regulate neuronal plasticity, survival and growth, alongside elevated translation and mRNA splicing pathways supporting enhanced gene expression and protein synthesis (Figure 5B). The reintroduction of *unc-25* in the neighboring glia rapidly promotes cellular clearance pathways such as aggrephagy and selective autophagy, crucial for clearing damaged proteins and maintaining proteostasis early in aging. Concurrently, pathways related to cytoskeleton organization and actin dynamics are upregulated, reflecting active remodeling and structural strengthening of neuronal circuits. Enhanced cytoskeletal remodeling and protein synthesis further reflect adaptive neuronal responses critical for recovery and maintenance. These results support prior findings that EGFR signaling promotes neuroplasticity and repair, while increased autophagy, including aggrephagy, aids in removing damaged proteins to protect neurons (Cochard et al., 2021; Haag et al., 2020).

In contrast, pathways associated with cellular stress responses such as FoxO, p53, mTOR, and MAPK signaling, along with processes like ribosome biogenesis and cell cycle regulation, were downregulated (Figure 5B). This may indicate an early-stage reduction in stress and proliferative signaling as neurons transition from an active remodeling state toward establishing a stable cellular environment. This stability is essential for initial neuronal adaptation and functional recovery following UNC-25 rescue.

By day 7, the transcriptomic profile shifts to emphasize long-term neuronal maintenance and stress resilience. The rescue condition shows increased activity in autophagy and lysosomal pathways, as well as enhanced ribosome function and translation, which reflect robust proteostasis necessary for neuronal health and homeostasis (Figure 5C). Additionally, upregulation of synaptic signaling components — including neurotransmitter clearance mechanisms, ion channel function, and chemical synapse pathways — indicates reinforcement of synaptic communication and ion homeostasis critical for proper neuronal signaling in aging neurons. These processes collectively support ongoing repair, removal of damaged cellular components, and fine-tuning of synaptic networks, helping neurons cope with cumulative stress and age-related deterioration. Conversely, key regeneration-related pathways such as axon regeneration, axon guidance, and neurogenesis, along with growth-related and stress-response signaling cascades including insulin, MAPK, and TP53 signaling are downregulated at this stage relative to wild-type (Figure 5C). This likely represents a strategic transition from the earlier phase of active regeneration observed at D1 toward a more stabilized phase focused on preserving established neuronal architecture and preventing further damage. The suppression of cellular senescence and metabolic pathways also suggests improved cellular health and reduced pro-aging signaling.

Overall, this balance between enhanced proteostasis, synaptic maintenance, and dampened regenerative/growth signaling reflects a shift toward cellular homeostasis and resilience that underpins the sustained neuroprotective effects of *unc-25* glial rescue in aging ASH neurons. Similarly, we previously observed decreased age-associated dendritic beadings in ASH neurons of the *unc-25* AMsh glial rescue mutant even after D7 as compared to wild-type, indicating neuroprotection and functional preservation linked to these transcriptional adaptations (Cheng et al., 2024).

**FIGURE 6:**
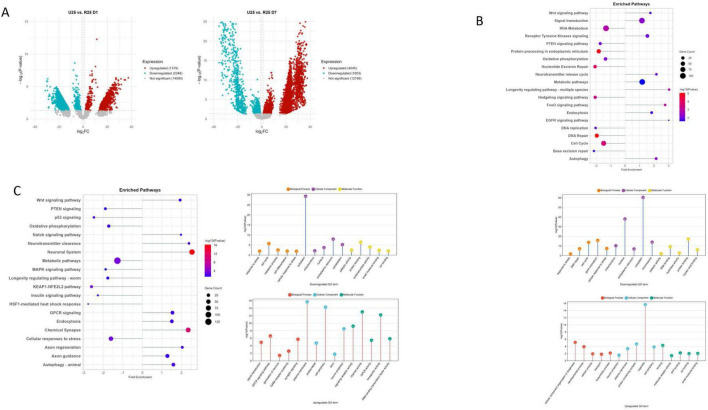
**(A)** Volcano plots of UNC-25 (U25) vs. UNC-25 rescue (R25) at day 1 (D1) and day 7 (D7). **(B)** Enriched pathways and GO terms for U25 vs. R25 D1. **(C)** Enriched pathways and GO terms for U25 vs. R25 D7.

Further, we examined the *unc-25* mutant and AMsh glial rescue to uncover a rapid activation of neuroprotective and signaling pathways at D1 (Figure 6A). Key pathways such as FoxO signaling, longevity regulating pathways, EGFR signaling, neurotransmitter release cycle, and receptor tyrosine kinase signaling are positively enriched, indicating that re-expression of *unc-25* in glia rapidly enhances intracellular trafficking, synaptic function, and cellular resilience mechanisms compromised in the mutant (Figure 6B). This transcriptional activation supports early neuroregenerative processes by promoting neuronal communication and adaptation. Concurrently, downregulation of pathways involved in DNA repair, cell cycle, oxidative phosphorylation, and protein processing in the endoplasmic reticulum suggests a shift away from stress and proliferative responses toward synaptic remodeling and functional specialization. These changes reflect an early remodeling phase where neurons prioritize restoring effective signaling and metabolic adjustments necessary for recovery.

By day 7, the transcriptomic profile of *unc-25* rescue shows a pronounced activation of pathways associated with synaptic maintenance, neuronal regeneration, and autophagy, alongside upregulation of Notch and Wnt signaling pathways (Figure 6C). These changes reflect enhanced synaptic function and remodeling, supported by increased activity in chemical synapse, neurotransmitter clearance, GPCR signaling, and GABA receptor clustering pathways, which collectively promote effective inhibitory neurotransmission and synaptic plasticity. The elevation of autophagy and endocytosis pathways highlights ongoing removal of damaged cellular components, sustaining neuronal integrity and resilience during aging. Conversely, the rescue condition exhibits downregulation of metabolic pathways, oxidative phosphorylation, and several stress response cascades, including the p53, MAPK, PTEN, insulin, and notably the HSF-1 mediated heat shock response pathway. Previously, we observed that targeted loss of HSF-1 specifically rescued age-dependent alterations in ASH neuronal calcium responses (Cheng et al., 2024). The decreased activation of stress-related pathways in the *unc-25* rescue condition corresponds with improved DNA damage response and synaptic structure regulation, emphasizing activation of repair and remodeling essential to counteract neurodegeneration.

Together, these dynamic transcriptional adaptations demonstrate that restoring GABA synthesis not only rectifies synaptic deficits but also mitigates age-associated cellular stress and excitotoxicity, fostering an environment favorable to neuronal survival and functional recovery during aging (Cheng et al., 2024). This underscores the pivotal role of glia-mediated GABAergic signaling in modulating both the progression of healthy neuronal aging and neuroprotection.

### 3.4 Dynamic shifts in Hedgehog signaling pathway

The Hedgehog signaling pathway is a fundamental regulator of numerous developmental processes and plays critical roles in maintaining neuronal health, particularly in the context of aging and neurodegenerative diseases (Dellovade et al., 2006; Yang et al., 2021). We observed a marked downregulation of Hedgehog signaling in both wild-type animals and *unc-25* mutants at D7 relative to D1, consistent with age-associated decline in regenerative and plasticity-related processes (Templeman et al., 2020). In wild-type animals, Hedgehog pathway downregulation is accompanied by reduced activity in pathways vital for metabolic support and cellular homeostasis, such as oxidative phosphorylation, metabolic pathways, ion homeostasis, MAPK signaling, as well as stress response and cellular senescence pathways. These changes reflect typical age-associated metabolic and signaling decline that may indirectly impair neuroregenerative capacity and neuronal function.

The downregulation of Hedgehog signaling in *unc-25* mutant co-occurs with suppression of pathways central to synaptic function and neuroprotection — including chemical synapse, GABA receptor activation, DNA repair, and cellular stress response pathways. This pattern suggests that loss of GABA synthesis amplifies deficits in mechanisms directly tied to neuronal resilience and regeneration. Hedgehog signaling decline is a common hallmark of neuronal aging; however, the associated patterns of pathway downregulation differ between genotypes (Dellovade et al., 2006; Shi and Murphy, 2023). While wild-type animals primarily exhibit age-related shifts in metabolic and homeostatic regulation, *unc-25* mutants — with impaired GABA synthesis — exhibit additional downregulation of synaptic function and neuroprotective pathways linked to GABAergic signaling. This distinction highlights how GABAergic dysfunction exacerbates declines in neural maintenance mechanisms, accelerating neuronal vulnerability during aging and linking Hedgehog signaling disruption to impaired inhibitory neurotransmission.

**FIGURE 7:**
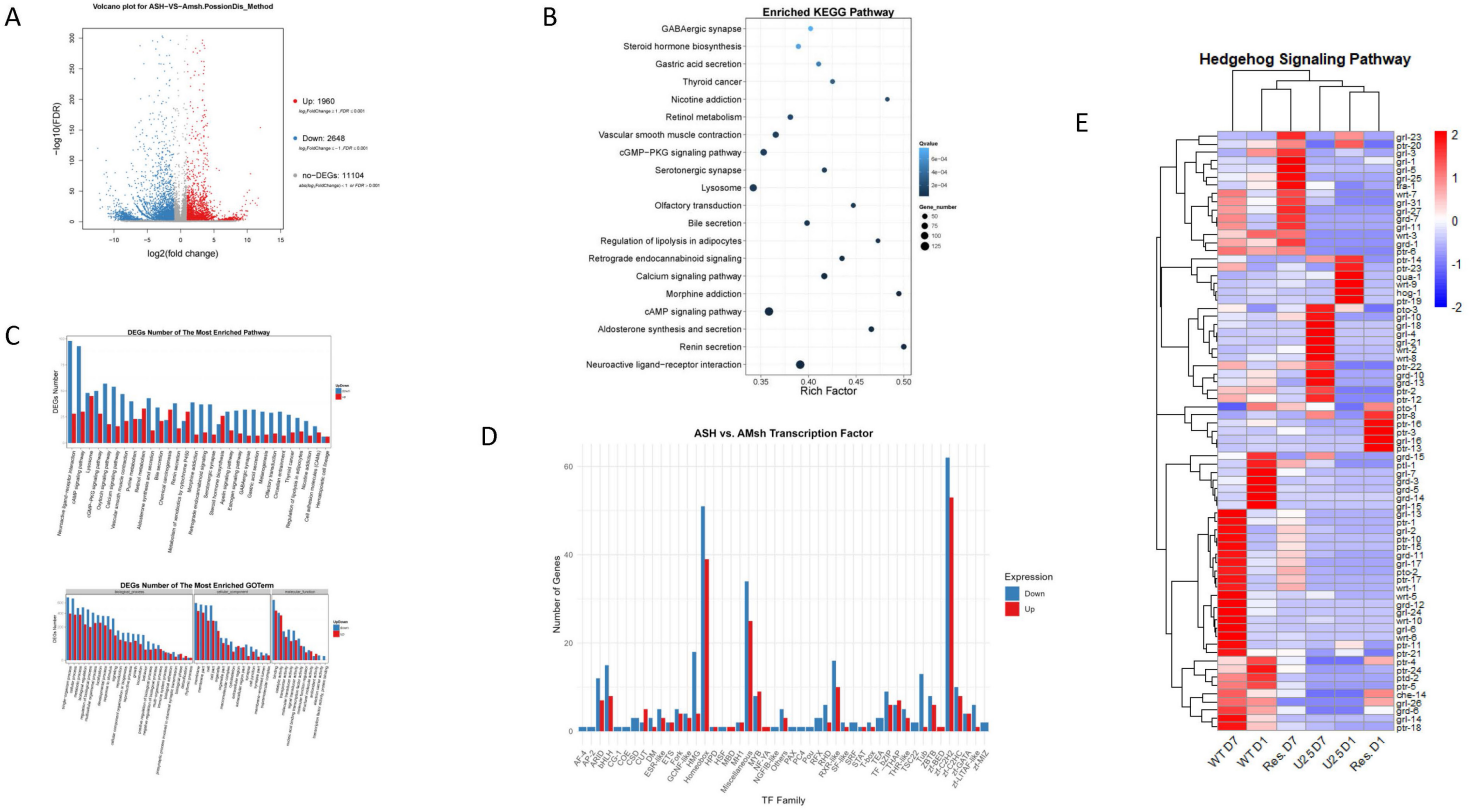
**(A)** Volcano plot of ASH neurons vs. AMsh glia. **(B)** Enriched KEGG pathway. **(C)** DEGs in Enriched Pathway and GO terms. **(D)** ASH vs. AMsh transcription factor. **(E)** Heatmap of Hedgehog signaling pathway genes.

In contrast, the rescue of *unc-25* expression specifically in AMsh glia results in a significant upregulation of Hedgehog signaling from D1 to D7 demonstrated by gene expression heatmaps showing a progressive increase in Hedgehog pathway components, such as TRA-1, most notably at D7 (Figure 7E). This activation coincides with enhanced expression of additional pathways associated with neuronal maintenance and longevity, such as DNA repair, insulin signaling, axon regeneration, and longevity-regulating KEAP1-NFE2L2 and p53 signaling pathways. The upregulation of metabolic pathways, ABC transporters, and PI3K-Akt signaling further reflects improved cellular homeostasis and stress resistance. These findings suggest that restoring GABAergic function in AMsh glia reengages a network of developmental, regenerative, and protective signaling cascades, thus promoting neuronal repair, plasticity, and healthy aging.

While *C. elegans* lacks the canonical Sonic Hedgehog (Shh) signaling found in mammals, it possesses a family of Hedgehog-related (Hh-r) proteins — including Warthog (WRT), Groundhog (GRD), Groundhog-like (GRL), and Quahog (QUA) — as well as Patched-related (PTR) receptors, which functionally parallel mammalian Hedgehog pathways in processes such as development, regeneration, and injury response, although acting through divergent mechanisms (Serra et al., 2024; Yang et al., 2021). These Hh-r and PTR proteins influence tissue structure, extracellular matrix formation, and may also have signaling roles contributing to neuronal maintenance during stress and aging (Serra et al., 2024). Dysregulation of either Hedgehog or GABAergic signaling pathways has been associated with increased susceptibility to neurodegenerative phenotypes and impaired neuronal repair across various animal models (Rallis et al., 2020; Yang et al., 2021). Notably, studies in rodent models have demonstrated that alterations in Sonic Hedgehog (Shh) signaling adversely impact the survival and functional integrity of GABAergic neurons (Delmotte et al., 2020; Rallis et al., 2020). Both signaling pathways have been implicated in the pathophysiology of neurodevelopmental disorders as well as in the progression of age-related neurodegenerative diseases.

Our gene expression heatmaps reveal dynamic regulation of specific Hh-r family members across aging and especially following the rescue of GABA synthesis in AMsh glia, supporting that Hedgehog-related signaling and GABAergic function are interconnected in maintaining neural plasticity and neuroprotection in aging nematodes (Figure 7E). Notably, TRA-1 is upregulated at D7 in the *unc-25* AMsh glial rescue condition. TRA-1 is the sole *C. elegans* ortholog of the GLI family of zinc-finger transcription factors and serves as a terminal effector of canonical Hedgehog signaling in other organisms (Mathies et al., 2004; Zeng et al., 2010). However, in nematodes, TRA-1 has been evolutionarily repurposed primarily as a master regulator of sex determination rather than performing the traditional Hedgehog developmental patterning roles (Berkseth et al., 2013). Despite the absence of canonical Hedgehog ligands and signaling components in *C. elegans*, TRA-1 retains essential regulatory functions beyond sex determination, including modulation of stress responses, transcriptional control of aging, and lifespan regulation (Ji et al., 2021). Importantly, TRA-1 enhances age-related pathways such as DAF-16/FOXO, contributes to stress resistance, and supports tissue-specific maintenance, particularly in neuronal populations (Ji et al., 2021; Tullet et al., 2014). The upregulation of TRA-1 in AMsh glial rescue at D7 suggests that restoration of GABAergic signaling activates TRA-1–dependent transcriptional programs, potentially linking sex determination and longevity pathways with neuroprotective mechanisms. This supports the role of TRA-1 as a key integrator of aging and stress response pathways, highlighting its pivotal function in promoting neuronal repair and healthy aging within the context of GABA-mediated glia-neuronal crosstalk.

Moreover, we observed a dynamic interplay between Hedgehog, Hippo, and Notch signaling pathways during neuronal aging and GABAergic dysfunction, with important implications for neuroprotection and glia-neuronal communication. In wild-type animals, Hedgehog signaling is downregulated from D1 to D7, consistent with age-associated decline, while Notch signaling and “Notch binding” terms are upregulated. This suggests that as Hedgehog signaling diminishes with age, Notch activation may compensate by supporting neuronal maintenance and plasticity to preserve neural homeostasis. In *unc-25* mutants lacking GABA synthesis, both Hedgehog and Hippo pathways are downregulated between D1 and D7, reflecting impaired neuroprotective and regenerative capacity. Restoration of GABAergic function in AMsh glia upregulates Hedgehog signaling, indicating that reestablishment of inhibitory neurotransmission reinvigorates critical regenerative programs. Notch signaling is also upregulated in rescued animals, implying coordinated regulation among these pathways to promote neuronal repair and healthy aging. Studies from *Drosophila*, zebrafish, and mammalian models suggests these signaling modules — Hedgehog, Notch, and Hippo — not only share downstream targets involved in neural stem cells and differentiation but also engage in reciprocal regulatory loops that are essential for neural plasticity and repair (Kong et al., 2015; Kumar et al., 2021; Ramos and Camargo, 2012). In addition, GABAergic signaling has been reported to interact with morphogen pathways such as Notch and Hedgehog, influencing neuronal fate specification and responses to neural injury, thereby supporting the integrated regulation of neural maintenance during aging and disease (Belgacem et al., 2016; Kong et al., 2015; Tran et al., 2025).

Together, our findings reveal a complex crosstalk among Hedgehog, Hippo, and Notch signaling pathways that respond distinctly to aging and GABAergic signaling. This interplay is critical for regulating adaptive neuroprotective mechanisms and glia-neuronal crosstalk, which maintain neuronal resilience and promote regeneration during aging. Notably, Hedgehog signaling can modulate Notch activity by influencing the expression of its ligands and receptors, while components of the Hippo pathway regulate the transcriptional outputs of both Hedgehog and Notch targets (Iluta et al., 2025; Jacobs and Huang, 2021; Ramos and Camargo, 2012). In *C. elegans*, the integration of these pathways — modulated by GABA — underscores a novel role for glial-neuronal interactions in orchestrating transcriptional programs essential for balancing neuronal maintenance, plasticity, and repair. This conserved signaling network highlights how inhibitory neurotransmission shapes the aging process and the regenerative capacity of neurons, offering promising avenues for therapeutic intervention against neurodegeneration.

### 3.5 Glia-neuronal crosstalk and GABA

We next performed comparative RNA-sequencing analysis of AMsh glia relative to ASH neurons of D1 young wild nematodes. This is to understand the specialized molecular mechanisms underlying AMsh glial modulation of ASH neuronal activity and characterize bidirectional glial-neuronal communication networks (Figure 7A and Supplementary Figures 1A–D, 2A–D). We observed a dynamic regulatory framework predominantly orchestrated by GABAergic signaling pathways, demonstrating the fundamental importance of GABA-mediated communication in sensory processing, synaptic homeostasis, and AMsh glia-ASH neuronal interactions (Figure 7B). The GABAergic synapse pathway emerged as one of the most significantly enriched pathways characterized by extensive downregulation of genes essential for inhibitory neurotransmission (Figure 7C; (Magnaghi, 2007). This is consistent with recent findings demonstrating that glial cells possess the ability to both respond to GABA through specific receptors and actively synthesize and release GABA to modulate adjacent ASH neuronal activity (Cheng et al., 2024; Yoon and Lee, 2014; Zhang et al., 2021).

The downregulation of neuroactive ligand-receptor interactions encompasses a broad spectrum of neuroreceptor genes, including metabotropic glutamate receptors, purinergic receptors, and neurotransmitter transporters. This particularly affects GABA receptor-mediated communication channels that are essential for effective signal transduction and the balance of excitatory and inhibitory signaling between neurons and glia (Cheng et al., 2024; Wang and Ho, 2023). Additionally, significant downregulation of transporter activity genes indicates reduced glial GABA transporter (GAT) function that fundamentally alters ambient GABA availability for glial-neuronal communication (Boddum et al., 2016). Extensive downregulation of calcium signaling pathway components represents critical disruption in bidirectional glial-neuronal crosstalk, as calcium responses in AMsh glia trigger GABA release to modulate ASH neuronal excitability (Duan et al., 2020; Wang L. et al., 2022).

The disruption of cAMP and cGMP-PKG signaling pathways compromises crucial secondary messenger systems that amplify neurotransmitter signals and regulate glial cell gene expression, metabolism, and morphology. Calcium-mediated gliotransmitter release from glial cells, which normally modulates synaptic transmission, is also affected (Augusto-Oliveira et al., 2020; Khakh and McCarthy, 2015). The downregulation of retrograde endocannabinoid signaling is particularly significant as this system enables sophisticated bidirectional neuron-glia communication. Unlike conventional neurotransmission, endocannabinoids travel backward from postsynaptic to presynaptic neurons, binding cannabinoid receptors to modulate synaptic strength and neuronal excitability (Carrera et al., 2020). This retrograde mechanism allows overactive postsynaptic neurons to suppress inhibitory GABA release through depolarization-induced suppression of inhibition (DSI), providing essential feedback control for neural circuit regulation (Barti et al., 2024).

Cellular component analysis reveals widespread structural differences between AMsh glia and ASH neurons at synaptic and cellular levels. Downregulation of membrane and synapse-related components indicates distinct molecular organization of specialized membrane domains where neuron-glia interactions occur (Figure 7C). Cell junction suppression is particularly significant since gap junctions form extensive networks enable rapid calcium wave propagation and metabolic coupling across the nervous system. This downregulation affects critical synaptic machinery, including protein scaffolds that organize neurotransmitter receptors, ion channels, and signaling molecules at neuron-glia interfaces. Reduced organelle expression suggests differential cellular metabolism and distinct capacity for energy-intensive neuron-glia communication processes. Conversely, upregulated extracellular region components reflect specialized functions or adaptive changes in extracellular matrix organization facilitating cell-cell communication.

The differential expression of transcription factors between AMsh glia and ASH neurons at D1 highlights distinct molecular profiles that underlie their specialized roles within the nervous system (Figure 7D). Notably, the C2H2 zinc finger and homeobox families exhibit strong regulation, emphasizing their critical involvement in neural fate determination and the modulation of sensory pathways. Other significant contributors include THAP domain proteins, which play important roles in neuroprotection and cell fate decisions, and bHLH factors that guide the differentiation processes of both neurons and glial cells. GATA factors are also balanced between up- and downregulation, suggesting important regulatory roles maintained across both lineages. Additionally, transcription factor families such as STAT and Forkhead participate in neuroprotective signaling pathways and facilitate glia-neuronal communication, while TF_bZIP members are implicated in managing cellular stress responses. Further contributors like ZBTB, MYB, ARID, RXR-like, and Tub families display diverse regulation, pointing to their involvement in chromatin remodeling, transcriptional plasticity, and lineage-specific gene programs. Collectively, these findings reflect a complex, tightly regulated network of transcriptional control that supports the distinct yet interdependent functions of glia and neurons, enabling dynamic interactions crucial for nervous system development and homeostasis.

The RNAseq analysis reveals a sophisticated molecular architecture of integrated neural networks dependent on precise neuron-glia communication, characterized by enriched and functionally specialized signal transduction pathways (Figures 7A–C). This pattern reflects intact cellular architecture with highly refined dynamic intercellular communication processes, extending beyond simple neurotransmission to broader neuroendocrine networks that integrate neural, glial, and systemic physiological responses across multiple systems in the wild-type organism (Allen and Lyons, 2018; Singhvi and Shaham, 2019). The observed differential expression of glia-neuronal communication pathways signifies the establishment of specialized cellular identities where distinct molecular mechanisms maintain bidirectional signaling balance. This crosstalk creates a dynamic equilibrium where downregulated AMsh glial functions are balanced by upregulated ASH neuronal responses, thus establishing a bidirectional compensatory mechanism for proper functions during the early adult stage (Fields and Stevens-Graham, 2002; Singhvi and Shaham, 2019). However, as nematodes age beyond D1, these compensatory mechanisms progressively deteriorate, disrupting glia-neuronal crosstalk and rendering neurons vulnerable to age-associated decline. Together, these findings underscore the critical role of GABAergic signaling as a key mediator in maintaining the delicate balance of bidirectional glia-neuronal crosstalk, which is vital for neural circuit stability and healthy neuronal aging (Cheng et al., 2024).

## 4 Discussion

Our comprehensive transcriptomic analysis of ASH neurons during aging in *C. elegans* reveals multiple interconnected molecular mechanisms governing neuronal decline and resilience, highlighting the pivotal role of GABAergic signaling mediated by adjacent AMsh glia. Consistent with conserved aging hallmarks across model organisms and humans, aging wild-type ASH neurons exhibited downregulation of genes involved in synaptic plasticity, mitochondrial function, and metabolic pathways, accompanied by compensatory upregulation of stress response and regenerative pathways. These underscore an intrinsic, yet insufficient, attempt by neurons to counteract functional deterioration through activation of neuroprotective programs and developmental pathways. This is consistent with prior reports that aging neurons enter a partially regenerative state but progressively fail to maintain homeostasis (Mattson and Arumugam, 2018; Navakkode and Kennedy, 2024).

Notably, we demonstrate that loss of GABA synthesis in *unc-25* mutants profoundly exacerbates the age-associated deleterious signatures. The deficiency of glial-derived GABA disrupted inhibitory neurotransmission and led to accelerated neuronal dysfunction marked by downregulation of pathways essential for synaptic maintenance and neuronal survival, alongside elevated stress and catabolic responses. This highlights the role of GABAergic signaling beyond synaptic inhibition, extending to the regulation of metabolic homeostasis and stress resilience. Similarly, such were observed in mammalian models where impaired GABAergic signaling contributes to excitotoxicity, mitochondrial distress, and calcium dysregulation during aging and neurodegeneration (Chun et al., 2015; Palop and Mucke, 2016; Verret et al., 2012).

The glial UNC-25 rescue, reinstating GABA synthesis specifically in AMsh glia, revealed a remarkable capacity for transcriptional reprogramming, which our transcriptomic data suggest is potentially associated with the restoration of neuronal homeostasis and viability. Notably, transcriptomic profiling of this rescue group identified downregulation of energy-intensive pathways (e.g., mTOR and oxidative phosphorylation), which may reflect potential metabolic conservation strategies, and upregulation of neuroprotective pathways including Hedgehog, p53, and PI3K-Akt signaling. This transcriptional signature echoes a putative shift toward enhanced neuronal maintenance, repair, and detoxification, supporting the notion that glial modulation of GABAergic signaling may act as a master regulator of neuronal resilience during aging, based on previously published studies (Boison and Steinhäuser, 2018; Jebelli et al., 2012; Tang et al., 2021). The rescue-induced transcriptional activation of axon regeneration and longevity pathways further indicates that glia-neuronal crosstalk may reinstate adaptive plasticity attenuated during aging, as implied exclusively by our transcriptomic observations. These transcriptomic findings point to potential associations between glial GABA restoration, warrant further functional validation in subsequent studies.

A key insight from our transcriptomic analyses is the dynamic regulation of the Hedgehog signaling pathway and its key transcriptional effector, TRA-1, following glial GABA rescue. Despite *C. elegans* lacking canonical Sonic Hedgehog components, our transcriptomic data hint at the possibility that the Hedgehog-related protein family and TRA-1 may mediate crucial neuroprotective and regenerative functions, potentially by integrating developmental signaling with stress response and lifespan pathways (e.g., DAF-16/FOXO), a regulatory pattern consistent with previously reported roles of these pathways. This aligns with findings in mammalian systems where Hedgehog signaling supports GABAergic neuron survival and function (Mathies et al., 2004; Rallis et al., 2020; Sun et al., 2017). Our transcriptomic data further suggest a potential interplay of Hedgehog, Notch, and Hippo pathways during aging—putatively influenced by GABAergic signaling, that may constitute a sophisticated regulatory network orchestrating neuroprotection and synaptic plasticity. This interpathway interaction is conserved across species and underpins neuronal longevity and repair, as supported by prior studies (Iluta et al., 2025; Ramos and Camargo, 2012; Zheng and Pan, 2019). The precise molecular mechanisms underlying these putative interactions remain unclear and merit in-depth functional investigations in future research, such as genetic manipulation and functional assays to validate the role of these pathways in glial GABA-associated neuronal aging resilience.

Our comparison of AMsh glia and ASH neurons delineates a refined molecular architecture of bidirectional glia-neuronal communication crucial for maintaining neuronal homeostasis. Aging disrupts this communication by altering gap junction expression and second messenger pathways, potentially weakening synaptic modulation and network integration. Glial GABAergic signaling is central in preserving this crosstalk, highlighting the therapeutic promise of targeting glia-derived inhibitory mechanisms to sustain synaptic integrity and neuronal function during aging (Allen and Lyons, 2018; Clarke and Barres, 2013).

Together, these findings construct a comprehensive model wherein glial GABA regulates neuronal aging through maintenance of excitation-inhibition balance, promotion of developmental and regenerative signaling pathways, modulation of stress responses, and preservation of neuron-glial communication. The ability of glial *unc-25* rescue to reverse adverse transcriptomic signatures and reactivate neuroprotective pathways underscores the plasticity of aging nervous system and points to glial inhibitory neurotransmission as a potent interventional target for neurodegenerative diseases.

Notably, the central role and key functions of GABAergic signaling are conserved across species, highlighting its translational relevance. In humans, GABAergic deficits are implicated in Alzheimer’s, Parkinson’s, and other age-related neurodegenerations (Kim and Yoon, 2017; Mele et al., 2019). Here, we showed that targeted glial GABA restoration rejuvenates neuronal signaling networks which opens new avenues for therapies aiming at glia-neuronal interactions and inhibitory signaling to mitigate neuronal decline and promote healthy brain aging (Cheng et al., 2024).

Our transcriptomic profiling suggests that glia-mediated GABAergic signaling may exert a critical influence on neuronal aging in *C. elegans*, with GABA loss associated with accelerated neurodegenerative-related transcriptomic changes and GABA restoration via AMsh glia linked to the activation of neuroprotective and regenerative pathways. The potential interplay of Hedgehog, Notch, and Hippo signaling cascades putatively modulated by GABA underscores a putative evolutionarily conserved mechanism that may integrate inhibitory neurotransmission with neuronal longevity and plasticity. These transcriptomic-derived insights highlight glial GABA as a compelling candidate therapeutic target worthy of further investigation for preserving neuronal health during aging and combating neurodegeneration.

## Data Availability

The datasets presented in this study can be found in online repositories. The names of the repository/repositories and accession number(s) can be found in the article/Supplementary material.
