# Supplementary material for: A transcriptomic resource for glial GABA-associated ASH neuronal aging and candidate pathways
Source: Front Aging Neurosci. 2026 Feb 23;18:1677754. doi: 10.3389/fnagi.2026.1677754 (PMC12968253; doi:10.3389/fnagi.2026.1677754)
Supplement: Supplementary file 1 [file Data_Sheet_1.pdf]

Supplementary Materials for

A transcriptomic resource for glial GABA-associated ASH neuronal aging and candidate pathways

Umar Al-Sheikh et al.

*Corresponding author. Umar Al-Sheikh, Email: ualsheikh@zju.edu.cn

*Corresponding author. Lijun Kang, Email: kanglijun@zju.edu.cn

*Corresponding author. Yongming Zhang, Email: zymcool@zju.edu.cn

The Supplementary Materials includes two supplementary figures.

Supplementary figures and figure legends

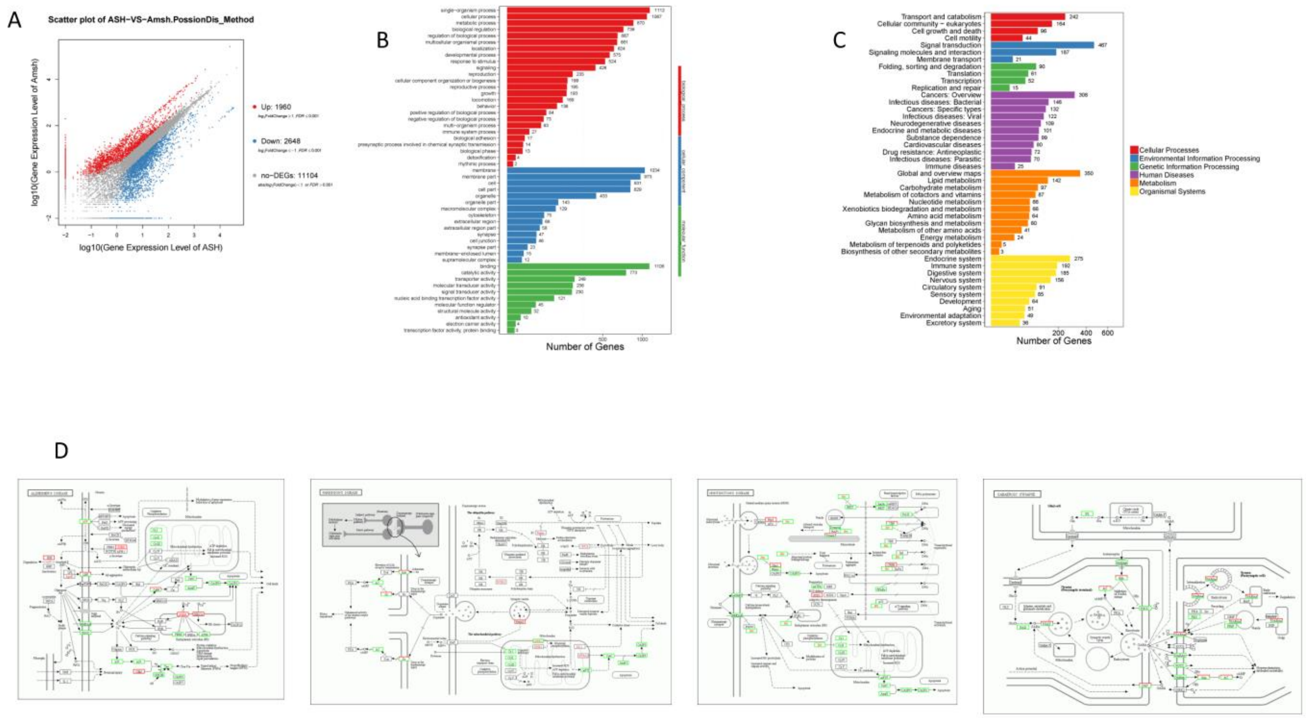

Supplementary Figure 1 (A) Scatter plot of ASH vs. AMsh. **(B)** Gene Ontology classification of DEGs. **(C)** Pathway classification of DEGs. **(D)** KEGG Pathway diagrams.

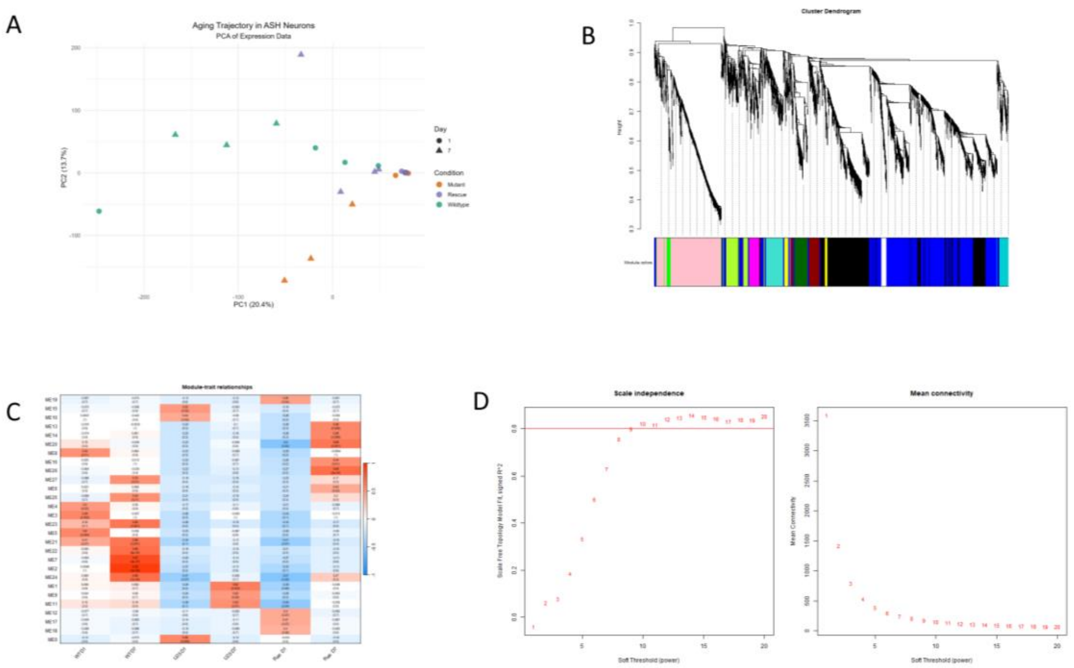

Supplementary Figure 2 (A) Principal component analysis (PCA) of the biological samples. **(B-D)** WGCNA (weighted gene co-expression network analysis) output. **(B)** Cluster dendrogram. **(C)** Module–trait relationship heatmap. **(D)** Soft-threshold selection plot.
